# Supplementary material for: Transcriptome Analysis Reveals Vimentin-Induced Disruption of Cell–Cell Associations Augments Breast Cancer Cell Migration
Source: Cells. 2022 Dec 13;11(24):4035. doi: 10.3390/cells11244035 (PMC9776984; doi:10.3390/cells11244035)
Supplement: Supplementary file 1 [file cells-11-04035-s001.zip › DEGs and enrichment analysis.zip!/DEGs and enrichment analysis/WT_VIMvsVector GO enrichment/ALL/WT_VIMvsVector_all.bar.pdf]

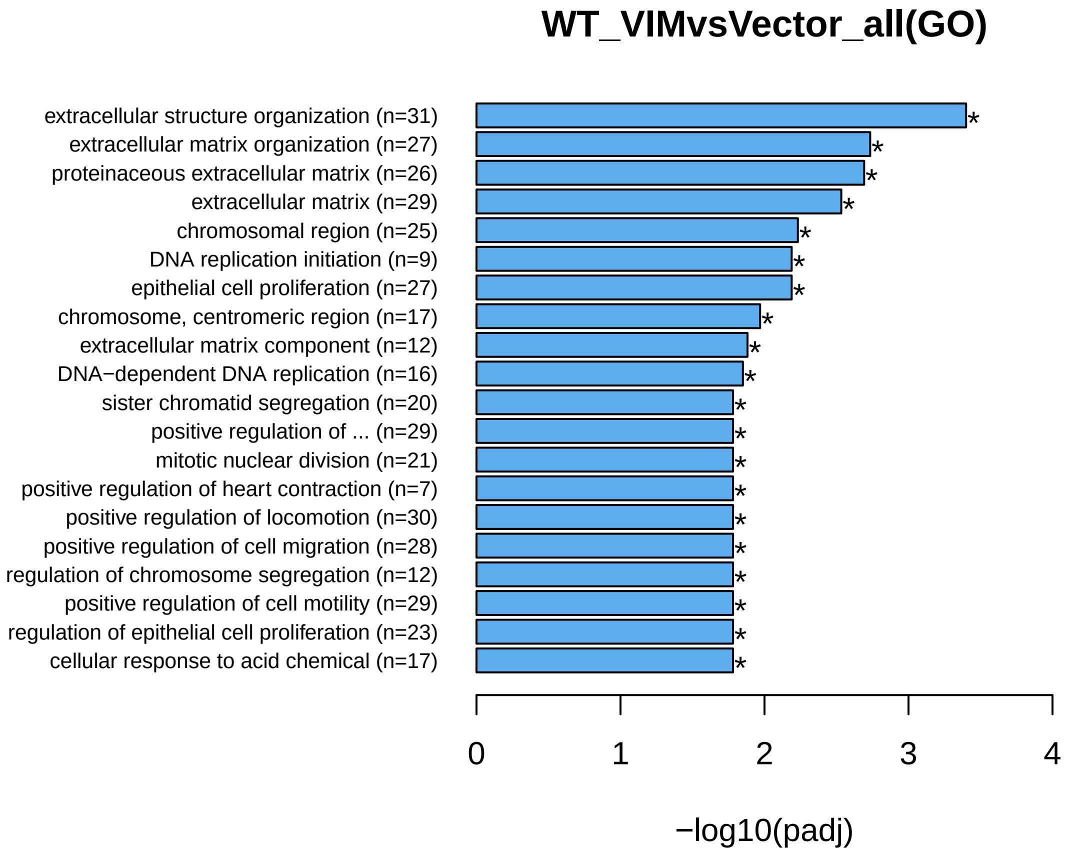
WT_VIMvsVector_all(GO)
extracellular structure organization (n=31)
extracellular matrix organization (n=27)
proteinaceous extracellular matrix (n=26)
extracellular matrix (n=29)
chromosomal region (n=25)
DNA replication initiation (n=9)
epithelial cell proliferation (n=27)
chromosome, centromeric region (n=17)
extracellular matrix component (n=12)
DNA−dependent DNA replication (n=16)
sister chromatid segregation (n=20)
positive regulation of ... (n=29)
mitotic nuclear division (n=21)
positive regulation of heart contraction (n=7)
positive regulation of locomotion (n=30)
positive regulation of cell migration (n=28)
regulation of chromosome segregation (n=12)
positive regulation of cell motility (n=29)
regulation of epithelial cell proliferation (n=23)
cellular response to acid chemical (n=17)
0
1
2
3
4
−log10(padj)
